# Streamlining pipeline efficiency: a novel model-agnostic technique for accelerating conditional generative and virtual screening pipelines

**DOI:** 10.1038/s41598-023-42952-y

**Published:** 2023-11-29

**Authors:** Karthik Viswanathan, Manan Goel, Siddhartha Laghuvarapu, Girish Varma, U. Deva Priyakumar

**Affiliations:** Center for Computational Natural Sciences and Bioinformatics, International Institute of Information Technology, Hyderabad, 500032 India

**Keywords:** Drug discovery, Computational science, Virtual drug screening, Machine learning

## Abstract

The discovery of potential therapeutic agents for life-threatening diseases has become a significant problem. There is a requirement for fast and accurate methods to identify drug-like molecules that can be used as potential candidates for novel targets. Existing techniques like high-throughput screening and virtual screening are time-consuming and inefficient. Traditional molecule generation pipelines are more efficient than virtual screening but use time-consuming docking software. Such docking functions can be emulated using Machine Learning models with comparable accuracy and faster execution times. However, we find that when pre-trained machine learning models are employed in generative pipelines as oracles, they suffer from model degradation in areas where data is scarce. In this study, we propose an active learning-based model that can be added as a supplement to enhanced molecule generation architectures. The proposed method uses uncertainty sampling on the molecules created by the generator model and dynamically learns as the generator samples molecules from different regions of the chemical space. The proposed framework can generate molecules with high binding affinity with $\sim$a 70% improvement in runtime compared to the baseline model by labeling only $\sim$30% of molecules compared to the baseline oracle.

## Introduction

Data-driven approaches have found profound success across multiple computer science domains—computer vision, natural language processing, signal processing, speech recognition, etc. These algorithms have made their way into drug discovery as well. With the increasing availability of open sources, well-curated datasets like ZINC^1^, ChEMBL^2^, and more have opened up avenues for using machine learning in different tasks like molecular property prediction, molecular structure prediction, retrosynthesis, and de novo molecule generation.

Conventionally, in order to identify potential molecules, high throughput screening (HTS) is performed on large databases in which every molecule in a database undergoes automated in vitro testing to find if they were a potential match^3^. High throughput screening is extremely expensive, inefficient, and has a low-hit rate^4^. This led researchers to formulate in silico computational approaches that could emulate protein-ligand interactions leading to high throughput screening experiments using physics-based property prediction methods to make the process more efficient^5^. Virtual screening is more cost-effective and efficient than high throughput screening. Further optimizations to virtual screening included clustering databases and using machine learning approaches to label molecules^6,7^. Given the linear search time, finding a desirable molecule becomes extremely ineffective when virtual screening is performed on huge databases. Moreover, the lack of chemical diversity in datasets has made virtual screening non-universal^8^.

The fundamental idea behind HTS and HTVS is to exploit the molecules already known in the chemical space but this number is infinitesimal in comparison to the estimated size of the chemical space with about $10^{60}$ synthesizable molecules^9^. Even the most exhaustive studies have been able to computationally evaluate only $10^{8}$ compounds^10^. Hence, the idea of de novo molecule generation emerged in which computational methods are used to generate molecules with certain properties. One way to achieve this is through genetic algorithms, which seek to evolve the ecosystem of pre-existing molecules into more desirable ones by introducing mutations in the current generation^11,12^. Unfortunately, genetic algorithms and their variants are prone to be stuck at local minima due to fixed initial populations and reverting mutations. To remove the initial requirement of a population set, deep generative models provide a vital improvement by forcing a non-linear relationship between molecular structures and properties^13^. In order to apply deep generative modeling to molecule generation, the two common representations are SMILES (simplified molecular-input line-entry system) strings and molecular graphs. SMILES strings possess their own grammar and semantics, and hence, this opens up the avenue for the application of natural language processing (NLP) based approaches like recurrent neural networks and transformers^14^. On the other hand, molecular graphs are generally heterogeneous graphs that can be used as an input to graph neural networks. Gupta et al.^15^ and Grisoni et al.^16^ used variants of recurrent neural networks to generate generic drug-like molecules while Bongini et al.^17^ and Mercado et al.^18^ used graph neural networks for molecule generation. However, drug molecules for a novel disease must possess a particular set of properties, and hence, methods were required to generate molecules with specified properties. This led to the application of more sophisticated generative models like variational autoencoders (VAE)^19^ and generative adversarial networks (GAN)^20^ along with optimization techniques like Bayesian optimization and reinforcement learning.

VAEs are capable of learning a continuous space representation of molecules which can then be optimized to get molecules with target properties through techniques like Bayesian optimization and swarm optimization. These techniques are architecture agnostic and can be applied with different forms of VAE like junction tree VAE by Jin et al., grammar VAE by Kusner et al. and more^21–24^. A VAE model was also paired with reinforcement learning for generating molecules with high binding affinity to a given target by Boitreaud et al.^25^ GANs are generative models that learn the probability distribution of the training data, and sampling from the distribution can then be used to generate synthetic data points. This model has also been applied to the generation of molecules with desirable properties in works by Cao et al., Prykhodko et al., Guimaraes et al. and Maziarka et al.^26–29^

However, the common theme across all the enhanced molecular generation models is an optimization algorithm requiring an oracle to calculate the property the model is being optimized for. Some properties are easy to calculate, while others, like binding affinity, take significantly longer. Molecular docking is a non-convex optimization problem and can take up to $\approx 10$ min for large molecules on a CPU. An alternative widely used is a machine learning predictor model that takes the molecule and target as an input and predicts the binding affinity^30,31^. However, this also comes with the caveat that the predictor model heavily depends on the initial training data, and hence, for molecule generation pipelines, as the model dynamically moves in the chemical space, the type of molecules being sampled also changes dynamically. This leads to a phenomenon called model degradation in which the performance of machine learning models declines as time passes^32^. Though faster, using machine learning models to predict binding affinity can become highly inaccurate as molecules start being sampled from regions of the chemical space unseen in the initial training set. Hence, there is a requirement for a predictor model that can: (1) perform the closest to that of physics-based docking software or a computationally demanding free energy calculation, (2) model which can work with a small dataset by learning posterior distribution accurately, and, (3) to develop a framework (over the machine learning model) that learns how to predict the binding affinity as the generator navigates through the chemical space.

Active learning is a popular technique in machine learning for training predictor models on datasets that are expensive to label. Bayesian active learning using uncertainty sampling was introduced in Computer Vision for Object detection^33^. More scalable and dynamic Active Learning approaches were introduced to improve training and network accuracy^34^. Active learning in cheminformatics was employed to conduct high throughput virtual screenings in existing databases^35–37^. Warmuth et al. uses support vector machines to mine data from an extensive collection of databases with ligands docked to a protein^38^. Raschka and Kaufman et al. summarize AI-based research for GPCR bioactive ligand discovery focusing on Active Learning^39^. Fujiwara et al.^40^ employ active learning using query by bagging to find structurally diverse hits in large databases. Gentile et al. conduct scalable AI-based virtual screening with deep docking^41^.

In this study, we propose a generative model agnostic active learning framework that can be used to accurately predict binding affinities throughout the optimization process of any generator model. The framework uses a Gaussian process regression model, updated at regular intervals using new training data obtained as the generator model is optimized to generate molecules with high binding affinity. This architecture was validated by integrating it with the MoleGuLAR pipeline proposed by Goel et al. It was found that using this reduced the training time by 70% while maintaining high accuracy^42^.

## Methods

**Figure 1:**
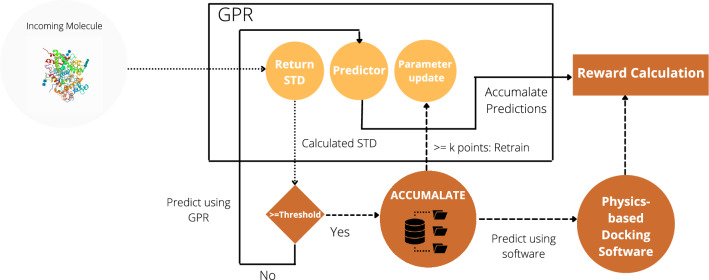
Architecture of the proposed dynamic predictor. An incoming molecule is used as input to a Gaussian Process Regression model which returns a prediction and uncertainty (standard deviation, STD). If the uncertainty is above a given threshold, the ground truth value of the property is calculated and added to a repository. If k points are accumulated in the repository, the model is re-trained, and the uncertainty threshold is updated.

This section describes the various components of the proposed dynamic predictor model, which can be used to replace the previously used slow docking tool and an inaccurate ML-based predictor model with an accurate ML-based predictor that learns new distributions with minimal data sampling. Figure 1 showcases the proposed predictor model using uncertainty-based active learning. Subsection *Gaussian process regressor* describes the formulation of Gaussian process regressor (GPR), the base predictor model. Initially, *k* points are sampled randomly from a database of drug-like molecules, their binding affinities with the target are calculated, and the predictor is trained to predict the binding affinity of these molecules with the required target. The GPR is updated dynamically using active learning, detailed in the subsection *Active learning*. The GPR also returns the uncertainty in its prediction, and the molecules used for retraining the model are picked using an uncertainty threshold which is also dynamic. Choosing this threshold is detailed in the subsection *Dynamic uncertainty threshold*.

### Gaussian process regressor

Gaussian process regressor (GPR) are the predictors used for molecular property prediction^43^. GPR calculates the probability distribution for fit over all possible functions that fit the data for a given distribution. When the GPR predicts the chemical property of a new incoming molecule, the prediction is tractable, and a normal distribution is obtained with mean and covariance. Hence, not only can a GPR return a prediction, but it can also return the uncertainty associated with that prediction in the form of standard deviation. In GPR, we first assume a Gaussian process prior *f*(*x*) using a mean function *m*(*x*) and covariance kernel function $k(x, x')$ represented by

1 $$\begin{aligned} f(x) \sim GP(m(x), k(x, x')) \end{aligned}$$

The prior assumes a multivariate distribution depending on the input dimensions. The mean function is usually a constant or the mean of the input dataset. The covariance function can be of any form of a function as long as it satisfies the properties of a kernel. The kernel function used for this study is the radial basis function (RBF) kernel^44^ represented by

2 $$\begin{aligned} k(x, x') = \sigma _{f}^{2} exp(\frac{-1}{2l^2}||x-x'||^2) \end{aligned}$$

with hyper-parameters: signal variance ($\sigma ^2$) and lengthscale (*l*). The GPR must be pre-trained with an initial set of points before inducting it into the Molecule generation pipeline. The input to the GPR for training is represented using SMILES. These smiles are converted into Mol2Vec embeddings^45^. These Mol2Vec embeddings are fed into a pre-trained GPR to predict the desired molecular property.

### Active learning

Active learning is a particular case of machine learning. Active learning algorithms can interactively query a database, acquire new data points, append them to the existing dataset, and retrain the current model. Active learning is used when unlabelled data is abundant, but labeling them is expensive and time-consuming - this is ideal. The function with which samples are acquired is known as the acquisition function. Common acquisition functions include balanced exploration, variance reduction, etc.

In this study, we employ Active learning using an uncertainty sampling-based querying strategy to acquire molecules whose properties are not predicted confidently by the ML model. When a set of molecules are to be evaluated by the oracle, the GPR initially predicts its uncertainty in predictions on each of these molecules. Depending on the Standard Deviation threshold, a molecule is deemed to be “certain” or “uncertain” with respect to the GPR’s prediction. Suppose the GPR is certain about the predictions of a molecule. In that case, the GPR makes the prediction, and the predictions are sent forward for the generative pipeline to calculate rewards. If the GPR is not certain about the predictions of a molecule, the molecule is stored in a repository. The molecule is then forwarded to a Physics-based property prediction software, and the results are then sent to the generative pipeline to calculate rewards. When *k* points are accumulated in the repository, these points are concatenated with the pre-trained points, and the GPR is retrained. This is done to learn the newly explored range by the generative pipeline for the associated molecular property. This step is performed repeatedly, and the number of retraining steps depends on the number of uncertain points encountered by the GPR.

### Dynamic uncertainty threshold

After every retraining step in the active learning pipeline, the model’s mean uncertainty for the regions it was initially trained on fluctuates. Hence, using a constant standard deviation threshold over multiple re-training steps can lead to inaccurate classification of a prediction as “certain” or “uncertain” concerning the ML model. Suppose the mean uncertainty of predictions of the pre-trained data during a retraining step is greater than the standard deviation threshold. In that case, the majority of the points are deemed uncertain and vice versa. Hence, it is only ideal for the standard deviation threshold to vary as the model’s mean uncertainty on the pre-trained data varies during every re-training step. To vary the threshold, a test set is maintained. After every retraining step, the uncertainties of the GPR on the new test set are recorded, and a histogram of the uncertainties is plotted after dividing the uncertainties into *k* bins. The uncertainties of the first bin are noted. Given the established benchmark (uncertainties belonging to the first bin), when trained on adequate data points for a given data range, the GPR can predict with lower uncertainty for data points belonging to any other successive bins. Hence, the new uncertainty threshold is the mean of the second bin.

### Dataset

**Figure 2:**
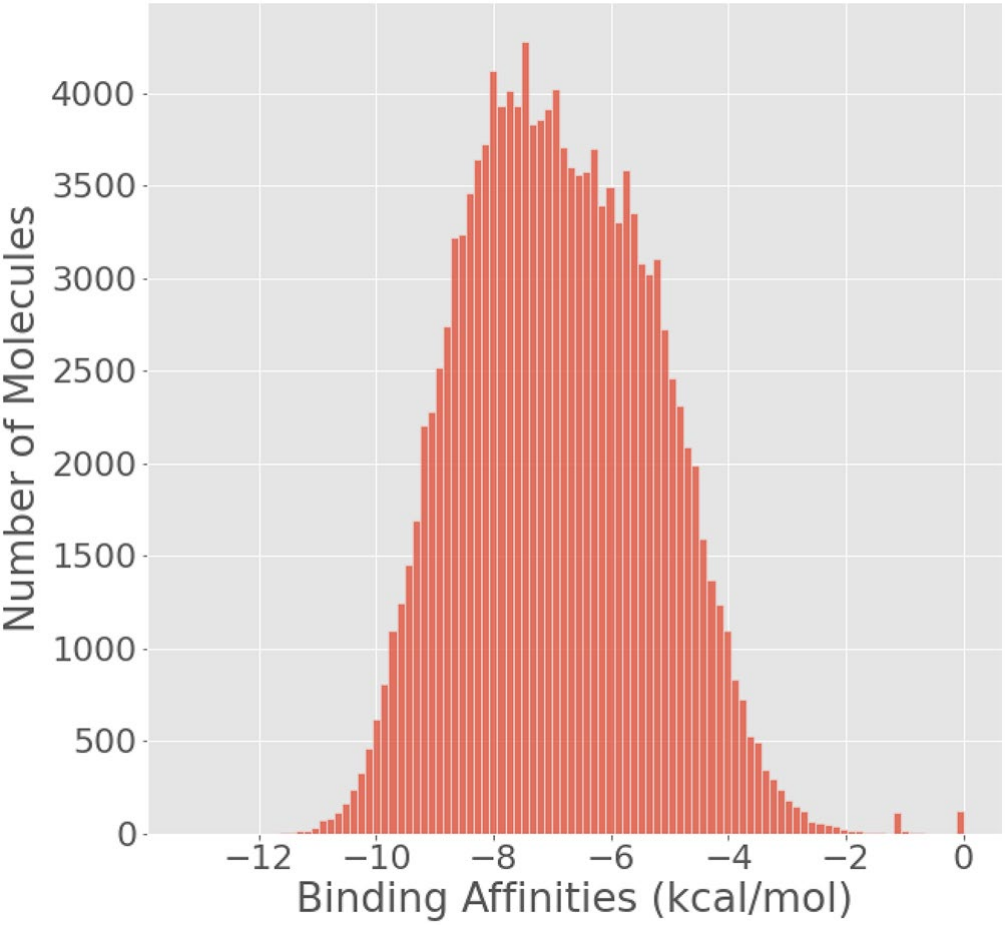
Distribution of binding affinities 150,000 molecules randomly sampled from the dataset. The dataset consists of $\approx$ 2 million molecules obtained from the HTS collection by Enamine^46^ docked with the TTBK1 protein.

The dataset used for this problem is the HTS collection by Enamine^46^. The dataset comprises approximately 2 million ligands, which were then docked to the Tau Tubulin Kinase 1 (TTBTK1), an important target for neurodegenerative diseases like Alzheimer’s^47^. Binding affinities in this dataset range from − 12 to 0 kcal/mol. The ground truth has been generated by docking the molecules using Autodock-GPU^48^. We follow the same docking methodology as Goel et al., and the molecules have been docked by following the same procedure for the 4BTK protein present in the **S6: Docking Methodology**, Supplementary Information for MoleGuLAR: Molecule Generation using Reinforcement Learning with Alternating Rewards^42^. For this problem, *k* points are randomly sampled from this dataset. Figure 2 depicts a histogram showing the distribution of the binding affinities of the molecules present in the dataset. As one can observe, the data gets more scarce in the higher binding affinity regions (regions < -8 kcal/mol). The scarcity of data in more negative binding affinity regions during virtual screening can be attributed to various factors. One reason is the presence of structural constraints and synthetic challenges associated with molecules that exhibit extremely negative binding affinities. Additionally, when utilizing a general-purpose database containing millions of ligands screened against a specific protein, it is possible that the chemical space explored within this database is more biased towards other target sets, resulting in limited coverage of the negative binding affinity regions. Furthermore, virtual screening alone cannot adequately explore newer chemical regions without the validation and input of a chemist who can suggest potential modifications for a set of promising molecules identified through virtual screening. These modifications, however, are often constrained by intellectual property considerations, which restrict the accessibility of data pertaining to higher binding affinity regions that hold greater potential for therapeutic applications. Hence, it is expected that a model trained on this data would have high error and uncertainty in the higher binding affinity regions.

## Results and discussion

**Figure 3:**
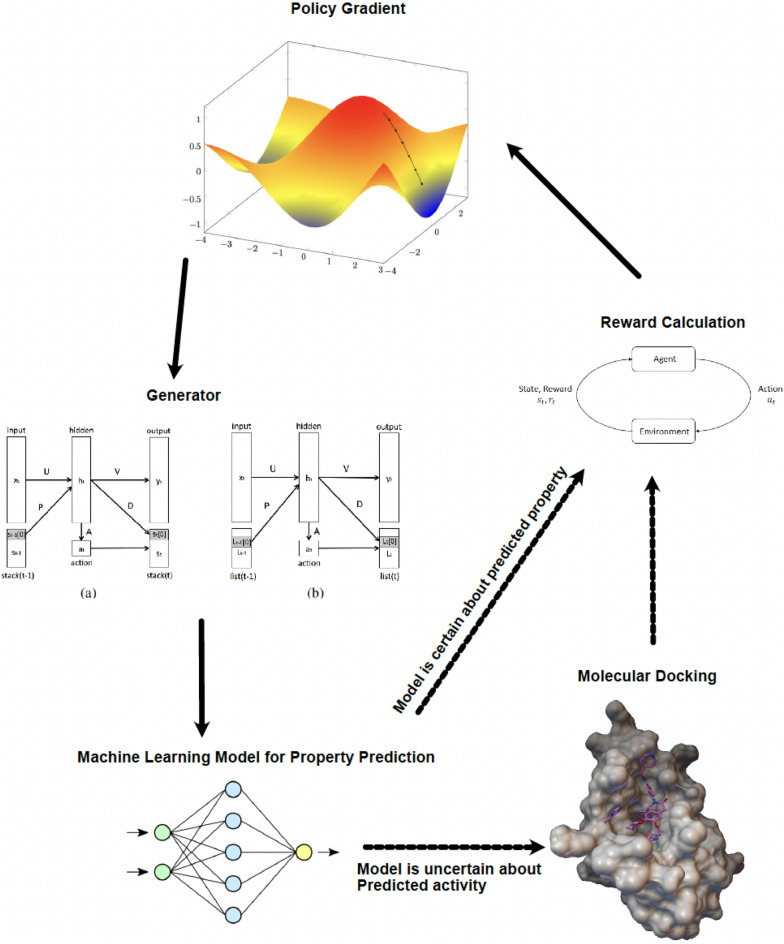
Active learning integrated with the MoleGuLAR pipeline.

This section reviews the results obtained by testing different parts of the proposed oracle represented in Figure 3. Subsection *GPRs for predicting binding affinity* discusses the performance of a GPR model trained to predict binding affinities to the TTBK1 protein. The following subsection compares two techniques for selectively labeling more data during molecule generation. Subsection *Active learning integrated with MoleGuLAR* explores how using the proposed enhanced predictor model improves the efficiency and accuracy of performing docking calculations and a conventional ML-based predictor model, respectively.

### GPRs for predicting binding affinity

**Figure 4:**
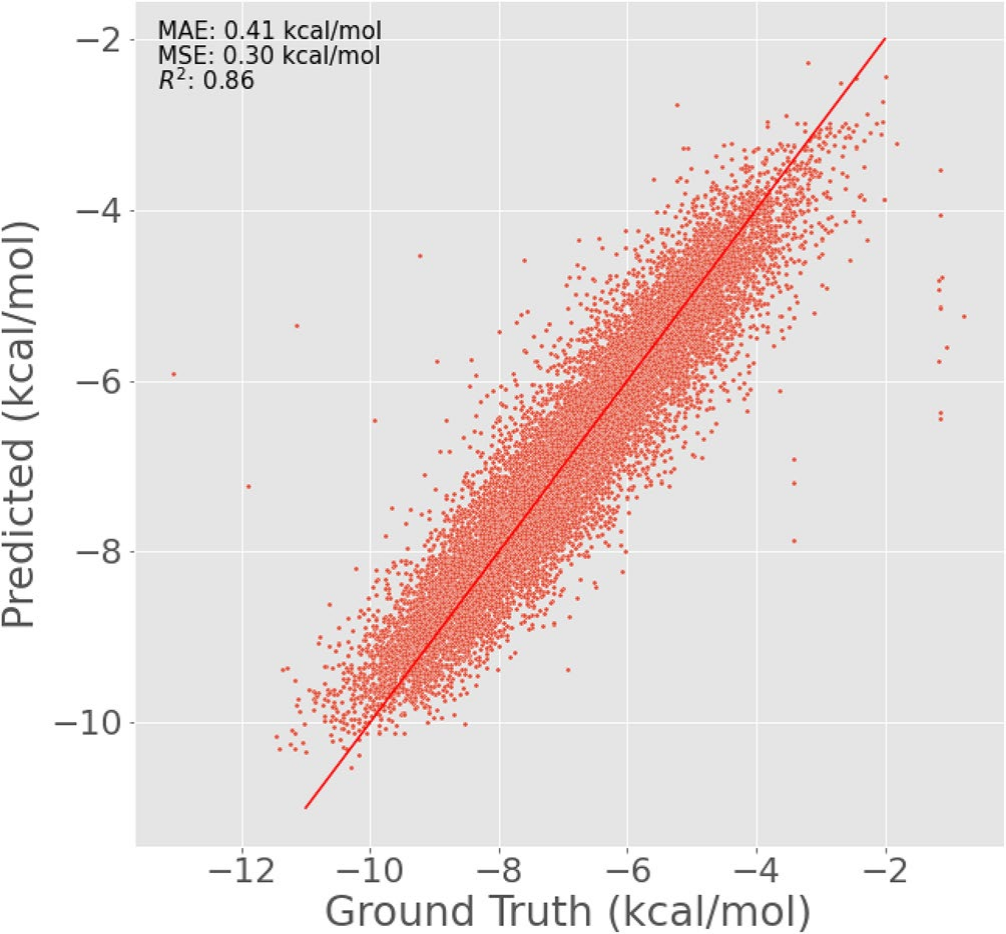
Correlation between ground truth and predicted binding affinities from a trained GPR model.

The GPR is trained on an initial data pool available from the Enamine dataset. Five thousand points are randomly sampled, and their Mol2Vec^45^ embeddings are extracted and trained using the following kernel: an additive RBF kernel^44^ with a length scale of 5.0 and White Noise kernel with default noise level (1.0). A 10,000-point test set is also sampled from the Enamine dataset to examine the accuracy of the dataset. The metrics achieved on the test set are: Mean Absolute error: of 0.452 kcal/mol, Mean squared error: of 0.378 kcal/mol, and R2 score of 0.87. Other models, including graph isomorphism networks, graph attention networks, and fully connected neural networks on Word2Vec embeddings, were tested. While fully connected neural networks performed the worst with an MAE of 1.2 kcal/mol, MSE of 2.56 kcal/mol, and R2 of 0.68, and GINs performed well with an MAE of 0.64 kcal/mol, MSE of 0.92 kcal/mol, and R2 of 0.74, Graph based models and other deep learning models required  70–80k points to achieve this accuracy. GPRs achieved benchmark accuracy with as low as 5000 data points. Our main goal in the optimization process is to explore newer chemical spaces more accurately and do so in less time, and labeling more points means employing the Physics-based property prediction software more—indicating an additional cost. Moreover, GPRs also provide us a better and more trivial estimation of uncertainty due to their probabilistic framework and incorporation of priors. Meanwhile, in the case of graph models and deep learning models, we used Monte Carlo Dropouts as approximations for deep Gaussian processes for estimating uncertainty $\sigma$, by extracting the variance across predictions. With sparse data inputs and lesser data points, along with shorter message passing in the case of small molecules meant that uncertainties fluctuated during every run. It was only with GPRs that we obtained a common trend where uncertainties were high in lesser explored regions of the dataset (<− 8 kcal/mol), and were low in data-abundant regions (<− 3 kcal/mol and >− 7 kcal/mol). Figure 4 represents the ground truth versus the predicted graph for the test set. This pool is the initially labeled dataset for the active learning problem.

### Active learning versus random sampling

**Figure 5:**
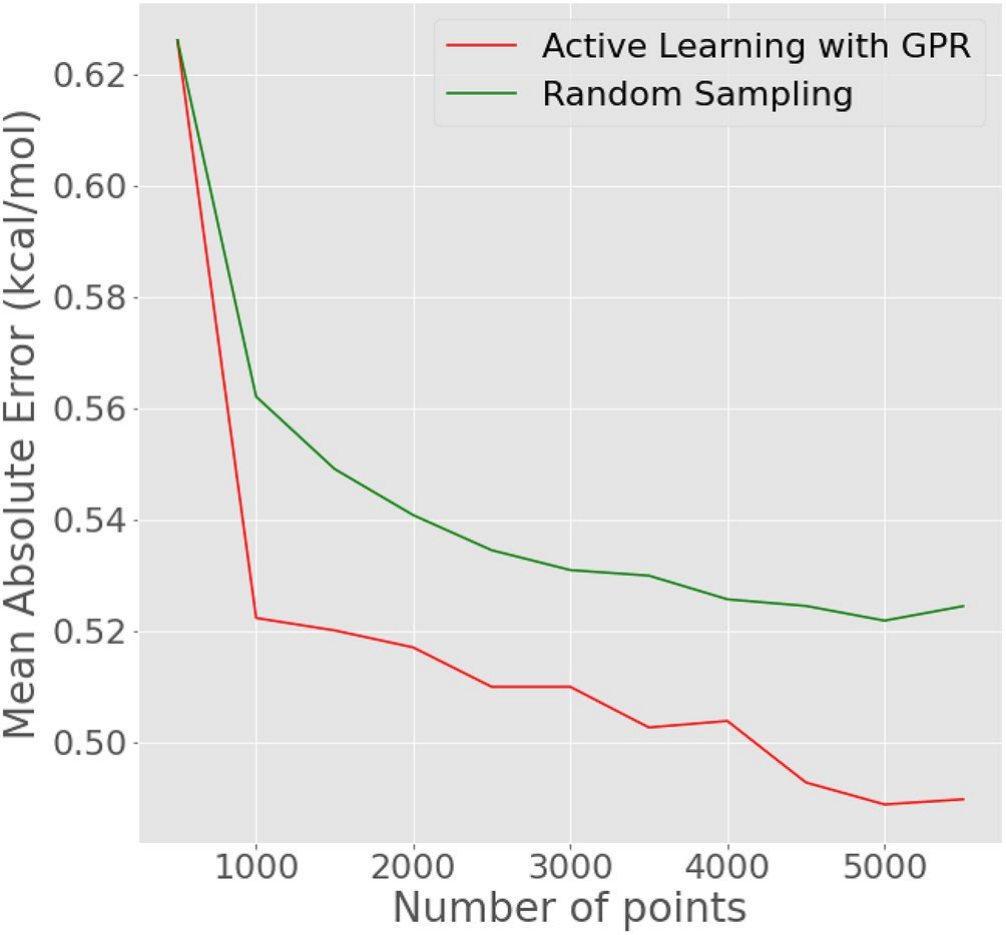
MAE versus Number of points in the training set. 500 Molecules from the Enamine dataset with the most uncertain predictions are appended to the training set and the GPR is retrained and the MAE is calculated on the hold-out test set.

As shown in the previous section, GPRs work well for predicting binding affinities to the given target, and the prediction uncertainty returned by the GPR can be leveraged to perform active learning. However, it is important to acknowledge the contribution of uncertainty sampling during chemical space exploration. To do so, we compare our uncertainty-based querying strategy with random sampling. The initial pool of training data consists of 500 data points from the enamine dataset. At every iteration, a GPR model is trained on the training data, and the 500 points from the entire Enamine dataset for which uncertainty is maximum are appended to the training set. Conversely, in the case of random sampling, the 500 points inducted into the training set are chosen at random. Figure 5 showcases the mean absolute error on a hold-out test set and shows that active learning outperforms random sampling and provides a better-performing predictor model.

### Active learning integrated with MoleGuLAR

#### Active learning versus random sampling

**Figure 6:**
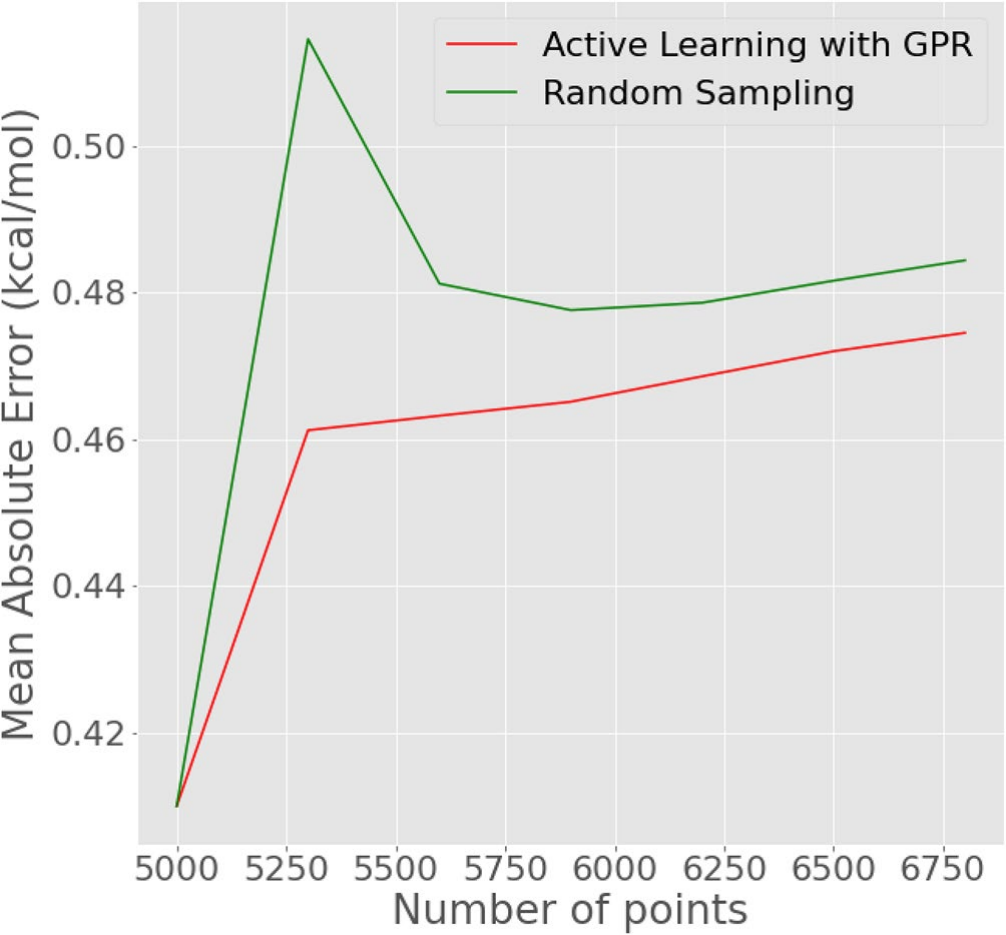
AL with GPR versus random sampling—Inside the RL Pipeline. 300 new points are obtained during each iteration and the model is re-trained. The MAE is calculated on the holdout test set at every retraining step.

The enhanced predictor model was integrated into the MoleGuLAR pipeline as the oracle. In MoleGuLAR, 500 molecules are generated to perform policy gradients and 100 more for evaluation during every iteration leading to 250 molecules in each iteration. The GPR makes a prediction for each of these molecules; for any molecule with uncertainty higher than the threshold, a docking calculation is performed, and the labeled molecule is added to a repository of molecules. As soon as the repository reaches a size of 300, these are inducted into the training set, the GPR is retrained, and the repository is reinitialized. Simultaneously, another repository is maintained for comparison in which randomly chosen molecules are inducted. Figure 6 compares the MAEs of the two GPRs with training data sampled using different strategies. The figure shows that the binding affinity increases as the number of points increases, which is the converse of what was seen in the previous section. This can be attributed to the generator model moving in previously unseen regions of the chemical space for which representation is low in the training data. It can also be noticed that at $\approx$ 6750 points, the difference gets significant. The sudden increase in mean absolute error (MAE) can be attributed to the RL framework’s initial exploration of new regions. During this exploration phase, the model encounters data from these regions, combined with a lack of uncertainty sampling, which hinders the accurate learning of distributions in the newly discovered space. As the gradients within the RL framework gradually decrease, the rate of exploration of new chemical spaces also slows down. Still, we see that the error in the case of Active Learning with GPR increases less steeply than random sampling and does not see large fluctuations.

#### Binding affinities of generated molecules

**Figure 7:**
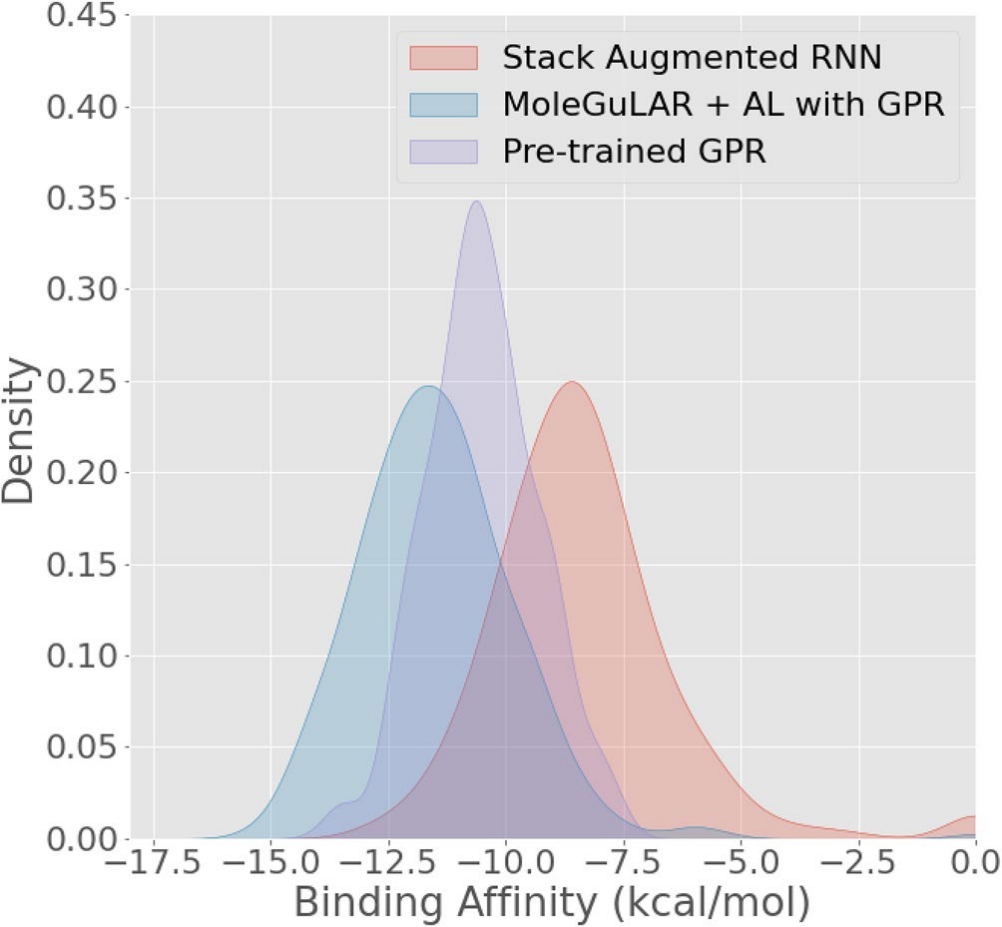
Distribution of binding affinities of molecules generated: using MoleGuLAR before optimization (red) and after optimization with pre-trained GPR (purple) and with GPR and active learning (blue).

To analyze and compare the “quality” of generated molecules based on the choice of Oracle, 500 molecules were generated using the generator before optimization. Following this, the generator was optimized for more negative binding affinity using a pre-trained static GPR as the oracle and the proposed dynamic predictor model as the GPR. At the end of the optimization process, 500 molecules are generated from both, and their ground truth binding affinities are calculated using AutoDock-GPU. The distribution of these binding affinities is shown in Figure 7. It is visible that using the proposed predictor leads to the generation of molecules with high binding affinities compared to using a static predictor. The reason for this is that static predictor has extremely poor quality predictions for molecules with high binding affinities and hence, fails to predict those values. Therefore, the proposed dynamic predictor model leads to better performance than a static predictor.

#### Course correction

**Figure 8:**
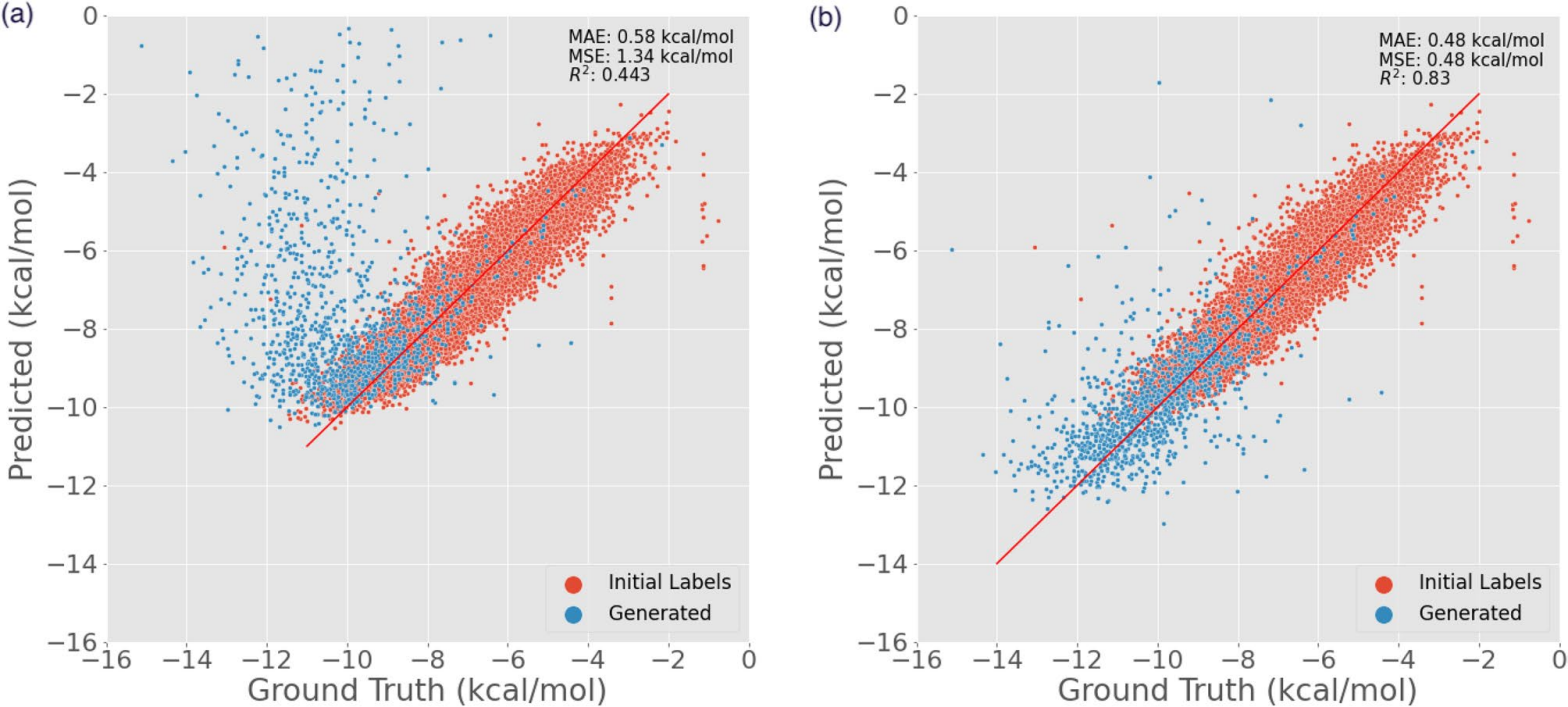
Course correction graph (**a**) before and (**b**) after re-training.

Analysis was also performed to check how the quality of predictions changes when the GPR model is retrained and whether it learns more information about the region of the chemical space being sampled. To do this, 1000 molecules were generated using MoleGuLAR after the optimization process, and their binding affinities were calculated using AutoDock-GPU. Predictions are then made using two models - the model used in the pipeline before retraining and after retraining. The correlation between these predictions and the ground truth values are present in Figures 8a,b, respectively. It is visible that the predicted binding affinities in regions where binding affinity $< -10$ kcal/mol are not close to the ground truth binding affinities. At the same time, after retraining, they lie much closer to the $y = x$ line. There is also a significant improvement in the *R*2 score and the MAE.

#### Improvement in efficiency

With evidence from the previous sections that the dynamic predictor model performs better than a static ML-based predictor model, the next step is to see if the accuracy trade-off by using a predictor model leads to a significantly shorter run time. Shorter run times not only promote cost-effectiveness but also provides researchers to run multiple models simultaneously under different parameters. We use Autodock GPU in all our docking calculations since Autodock GPU provides us with an accelerated framework for docking calculations. With Autodock-Vina, on average, one molecule would take  20 s to dock. With Autodock GPU, we are able to accelerate this process to  3–4 s per molecule. We compare the time taken by three different versions of MoleGuLAR under three different settings: the base model proposed by Goel et al., the pre-trained model proposed by Goel et al., and our active learning framework. The time taken to optimize MoleGuLAR for 100 iterations and the total number of docked molecules is presented in Tables 1 and 2, respectively.

**Table 1:** Time analysis: MoleGuLAR.

Oracle	Time
Autodock-GPU	43 h
Active learning	13 h
Pre-trained	8 h

**Table 2:** Labeling Analysis: MoleGuLaR.

Oracle	Number of points labeled inside / outside the pipeline
Autodock-GPU	25000 / 0
Active Learning	2600 / 5000
Pre- trained	0 / 5000

It is evident that in terms of time taken the pretrained model is the most efficient but it makes bad predictions. On the other hand AutoDock-GPU makes accurate predictions but can take up to 2 days and hence, is extremely time consuming. The pre-trained model labels 20% of total molecules compared to the number of molecules generated by the pipeline. The active learning also labels 20% outside the pipeline—just like the pre-trained model, but also labels an additional 10.4% inside the pipeline. Hence, the active learning-based predictor finds a balance between the quality of predictions and the time taken.

## Conclusion

In this study, a solution is presented to make the de novo generation of drug-like molecules more efficient. Active learning and uncertainty sampling are used to reduce the execution time of molecule generation pipelines. The approach is validated by conducting rigorous experiments which test the accuracy and the correctness of the Active learning pipeline and showed that Active learning acts as a trade-off between complete docking and a pre-trained Machine learning model which explores a local non-linear function to learn about the binding pocket. We also show that the trade-off proves to be very important in improving the accuracy and the distribution of the predicted molecules in the pre-trained model. Further work can include reducing the number of labeled points to a greater extent and altering graph-based machine learning models to work with smaller datasets. But, using a simplistic base model in this problem significantly improves the execution time and reduces the number of docking calculations.

## Data Availability

The data, code, analysis, models and the generated molecules have been included at https://github.com/devalab/Enhanced-MoleGuLAR.
